# Optimizing Irrigation and Nitrogen Inputs for Balancing Greenhouse Gas Mitigation, Productivity, and Profitability in an Intercropping System of Wolfberry and Alfalfa

**DOI:** 10.3390/plants15132038

**Published:** 2026-07-01

**Authors:** Junkui Jia, Boda Li, Yuanbo Jiang, Huile Lv, Yaya Duan, Yanbiao Wang, Jinxi Chen

**Affiliations:** 1 Gansu Province Jingtaichuan Electric Pumping Irrigation Water Resources Utilization Center, Baiyin 730400, China; l156158114@163.com; 2 College of Water Conservancy and Hydropower Engineering, Gansu Agricultural University, Lanzhou 730070, China; 1073323010121@st.gsau.edu.cn (Y.J.); 1073323020364@st.gsau.edu.cn (H.L.); 1073323020376@st.gsau.edu.cn (Y.D.); 1073323020378@st.gsau.edu.cn (Y.W.); 1073324120804@st.gsau.edu.cn (J.C.); 3 Gansu Province Agricultural Intelligent Water-Saving Technology Innovation Center, Lanzhou 730070, China

**Keywords:** soil environment, greenhouse gas flux, intercropping system, global warming potential, comprehensive evaluation

## Abstract

Water and nitrogen management influences farmland productivity and greenhouse gas emissions by regulating the soil micro-environment. However, the synergistic optimization strategy among yield improvement, economic benefit, and emission reduction in intercropping systems in arid regions remains unclear. Based on a two-year field experiment using an intercropping system of wolfberry and alfalfa, this study established four irrigation levels [full irrigation (W0), mild water deficit (W1), moderate water deficit (W2), and severe water deficit (W3)] and four nitrogen application levels [0 (N0), 150 (N1), 300 (N2), and 450 kg·ha^−1^ (N3)]. The effects of water and nitrogen regulation on soil hydrothermal conditions, greenhouse gas emissions, crop yield, and economic benefits were systematically analyzed. The results showed that soil water content increased with higher nitrogen application rates but decreased with a more severe water deficit. In contrast, soil temperature exhibited the opposite trend, with the W3 treatment increasing by 2.23–2.41 °C compared to W0 during the full fruiting period. The emission fluxes of CO_2_ and N_2_O increased with higher nitrogen application rates but decreased with a more severe water deficit. CH_4_ acted as a sink, with its uptake decreasing as nitrogen application increased and the water deficit intensified. CO_2_ was the dominant contributor to the global warming potential of the intercropping system of wolfberry and alfalfa, accounting for 85.3–94.6% of the total. The emission fluxes of CO_2_ and N_2_O were significantly positively correlated with the soil water content, while the CH_4_ emission flux was significantly positively correlated with the soil temperature. The W0N2 treatment achieved the highest system yield and net profit, whereas the W1N2 treatment exhibited the highest return on investment. A comprehensive evaluation using the entropy weight–TOPSIS model identified W1N2 as the optimal treatment. An integrated water–nitrogen decision model determined that the optimal water and nitrogen combination for achieving a high yield, a high efficiency, and low emissions was an irrigation amount of 4245–4413 m^3^·ha^−1^ and a nitrogen application rate of 290–323 kg·ha^−1^. The findings of this study can provide a scientific basis for the sustainable water and nitrogen management of characteristic cash crop intercropping systems in arid regions.

## 1. Introduction

Balancing food security, ecological protection, and efficient resource utilization is central to achieving sustainable agricultural development in the new era. This is not only a core objective of the United Nations 2030 Agenda for Sustainable Development—specifically the goals of ending hunger and taking climate action—but also a key priority of China’s Strategic Plan for Accelerating the Construction of an Agricultural Powerhouse (2024–2035) and its “carbon peak and carbon neutrality” strategy [1]. As key regions for China’s green agricultural development, arid and semi-arid areas face challenges such as water scarcity and low soil nutrient levels [2]. Irrational water and nitrogen inputs have led to an imbalance in the carbon and nitrogen cycles within the soil–crop system, which not only constrains regional crop productivity but also increases greenhouse gas (GHG) emissions from farmlands [3]. Therefore, developing a water and nitrogen regulation model that ensures both an increased crop yield and farmer income while effectively suppressing GHG emissions in arid and semi-arid regions has become an urgent issue for China’s green agricultural transition.

As a typical ecological cropping pattern, forest–grass intercropping plays an important role in optimizing cropping structures, promoting crop–livestock integration, and improving overall farmland productivity [4]. In recent years, researchers have conducted extensive studies on water regulation and the ecological effects of forest–grass intercropping systems. The results show that forest–grass intercropping improves soil quality in coastal saline–alkali habitats [2]; it significantly increases the soil organic matter content in the 0–20 cm surface layer and reduces the soil bulk density [5]. Forest–grass intercropping also significantly enhances the land equivalent ratio (LER), improves the soil physical properties, and increases the soil water content, electrical conductivity, total nitrogen, and available phosphorus [6]. It notably affects soil organic carbon fractions and the activities of most carbon–nitrogen (C–N) cycling enzymes. Compared with monocropping, forest–grass intercropping increases the soil organic carbon (SOC), total nutrients, and available nutrients by 27.3% to 55.3% [4]. Mild water regulation combined with intercropping increases the root length density (RLD), leaf area index (LAI), cumulative intercepted photosynthetically active radiation (CIPAR), and radiation use efficiency (RUE) in forest land [7]; mild water regulation also effectively improves the yield and quality of forest–grass intercropping systems [8].

The current research on forest–grass intercropping has mostly focused on physiological and ecological characteristics and soil improvement effects. Less is known about how water and nitrogen management simultaneously shapes the soil hydrothermal environment, regulates GHG emissions, and determines the crop yield in these systems. Furthermore, there is a lack of quantitative evaluation and optimal management thresholds that balance “high yield and high efficiency” with “low carbon emissions.” The Gansu Yellow River Irrigation Zone, located in the upper reaches of the Yellow River, is the second-largest comprehensive agricultural commodity production base in Gansu Province. Nevertheless, this region faces prominent issues such as the risk of secondary soil salinization, low water use efficiency, and severe nitrogen loss, which have become major constraints to the green and high-quality development of regional agriculture. *Lycium barbarum* (wolfberry) and alfalfa are highly tolerant to saline–alkali conditions and environmental stresses. Intercropping the two species can enhance land use efficiency and ecosystem services through complementary deep and shallow root systems, synergistic canopy light and heat utilization, and biological nitrogen fixation by alfalfa rhizobia. The intercropping of wolfberry and alfalfa provides ecological benefits through complementary root systems and biological nitrogen fixation by alfalfa, which can reduce the fertilizer input and improve the soil quality. Therefore, this study focuses on the intercropping system of wolfberry and alfalfa in the Gansu Yellow River Irrigation Zone, with the following objectives: (1) to elucidate the characteristics of the soil hydrothermal environment and GHG emissions under water–nitrogen regulation; (2) to reveal the interrelationships between the soil hydrothermal environment and GHG emissions; and (3) to propose a regionally suitable water and nitrogen management model that balances yield, economic benefits, and emission reduction.

## 2. Results

### 2.1. Soil Water and Thermal Environment

#### 2.1.1. Soil Water Content

**Figure 1:**
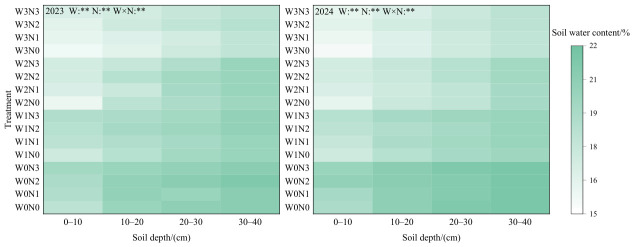
Effects of water and nitrogen regulation on soil moisture content in the intercropping system of wolfberry and alfalfa. W, N, and W × N represent the main effects of irrigation gradient, the main effects of nitrogen application level, and their interaction effects, respectively, ** indicates significance at the *p* < 0.01 level.

The irrigation gradient, the nitrogen application level, and their interaction had significant effects on the soil water content in each soil layer of the intercropping system of wolfberry and alfalfa (Figure 1). Overall, the soil water content increased with soil depth, and the differences among treatments were mainly concentrated in the surface layer, gradually diminishing with increasing depth.

During the whole growth period, under the same irrigation condition, the N2 treatment exhibited the highest soil water content, significantly higher than those of the N0 and N1 treatments. Under the same nitrogen application level, the soil water content decreased with a reduced irrigation amount. Under the N0 condition, the W1, W2, and W3 treatments decreased by 4.85%, 8.46%, and 14.80% lower than that in the W0 treatment, respectively. Under the N1, N2, and N3 conditions, deficit irrigation reduced the soil water content by 16.60–20.71%, 16.62–21.10%, and 16.90–20.82%, respectively, compared with full irrigation. In the 10–20 cm soil layer, the treatment differences were the largest, with the W0N1 and W0N2 treatments reaching a water content of 20.3%, which was 3.70 percentage points higher than that of the W3N1 treatment. In the 30–40 cm soil layer, the W0N2 treatment had the highest water content (21.10%), which was 16.57% higher than that of the W3N0 treatment. The trend in 2024 was generally consistent with that in 2023, but the differences among soil layers showed slight variations: in the 0–10 cm layer, the differences among treatments were most pronounced; in the 10–20 cm layer, the W0N3 treatment had the highest water content (20.70%); and, in the 30–40 cm layer, the W0N2 treatment remained the highest (21.50%).

#### 2.1.2. Soil Temperature

**Figure 2:**
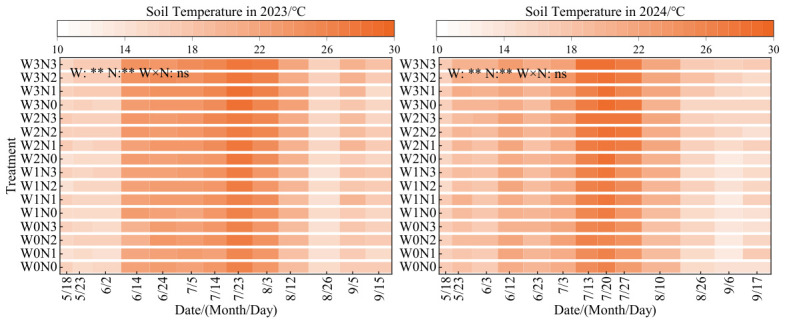
Effects of water and nitrogen regulation on soil temperature in the intercropping system of wolfberry and alfalfa. W, N, and W × N represent the main effects of irrigation gradient, nitrogen application level, and their interaction, respectively; ** indicates significance at the *p* < 0.01 level; and ns indicates no significance.

A two-way ANOVA revealed that both irrigation (W) and nitrogen application (N) had significant effects on soil temperature (*p* < 0.01), while their interaction (W × N) was not significant (*p* > 0.05). During the growth periods of 2023 (14.1–26.79 °C) and 2024 (13.01–27.97 °C), the trends in soil temperature were generally consistent, both exhibiting a unimodal curve (Figure 2). In the early growth stage (May), the soil temperature gradually increased with rising air temperature. In May 2023, soil temperatures across treatments ranged from 14.23 °C to 16.89 °C. In May 2024, overall temperatures were higher than those in 2023, with a minimum of 15.08 °C and a maximum of 20.00 °C. During the full fruiting stage (late July), the soil temperature reached its peak for the entire growth period. In 2023, the maximum temperature occurred around 23 July, with the W3N0 treatment recording the highest value (28.63 °C), and the average temperature across all treatments was 27.10 °C. In 2024, the maximum temperature occurred around 20 July, again with the W3N0 treatment being the highest (29.31 °C), and the average temperature across all treatments was 27.54 °C. After August, the soil temperature gradually decreased.

Under the same nitrogen application level, the soil temperature decreased with the increasing irrigation amount. The average soil temperature over the whole growth period followed the order: W0 (19.55 °C) < W1 (19.83 °C) < W2 (20.51 °C) < W3 (21.20 °C). The differences were particularly significant during the full fruiting stage. In 2023, the average temperature of the W3 treatment was 25.43 °C, which was 2.23 °C higher than that of the W0 treatment (23.20 °C). In the same period of 2024, the average temperature of the W3 treatment was 26.09 °C, which was 2.41 °C higher than that of the W0 treatment (23.67 °C).

### 2.2. Soil Greenhouse Gas Emissions

#### 2.2.1. Greenhouse Gas Emission Flux

(1) CO_2_ Emission Flux

**Figure 3:**
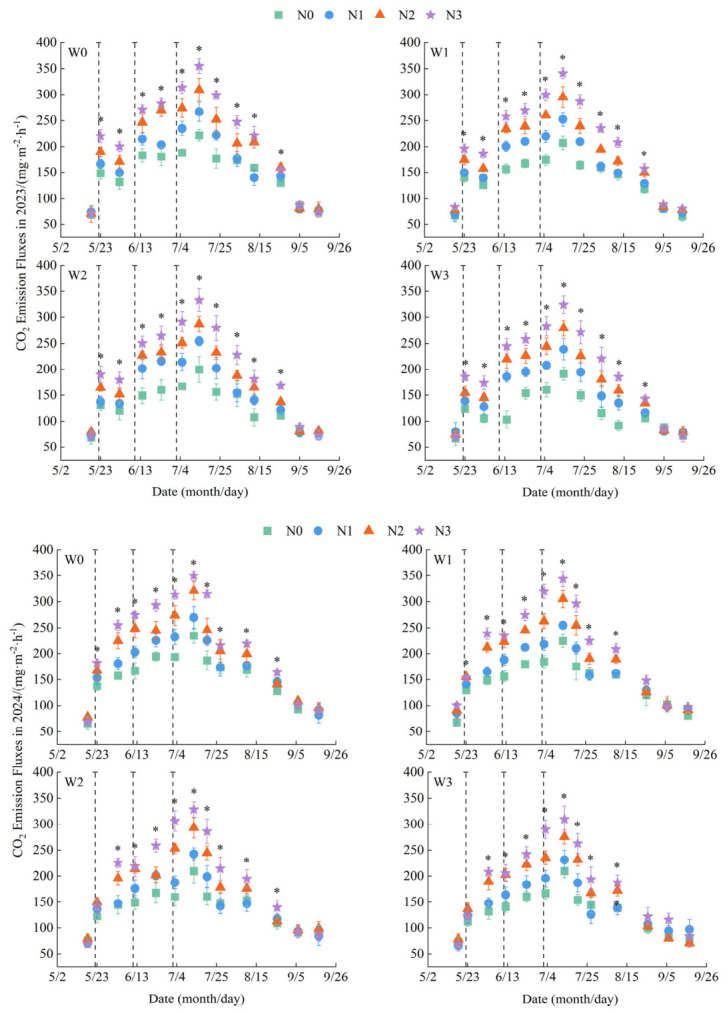
Effects of water and nitrogen regulation on CO_2_ emission flux in the intercropping system of wolfberry and alfalfa in 2023~2024. * indicates extremely significant differences among nitrogen application levels under the same irrigation gradient (*p* < 0.01), and dotted lines indicate the timing of nitrogen application (along with irrigation) treatments.

Throughout the entire growth period, the soil of the wolfberry field acted as a source of CO_2_ emissions, with the emission flux exhibiting a seasonal “single-peak curve” that peaked in mid-July (Figure 3). The CO_2_ emission flux increased significantly with higher nitrogen application rates and decreased with a more severe water deficit. Among all treatments, the W0N3 treatment had the highest CO_2_ emission flux, averaging 193.67–215.15 mg·m^−2^·h^−1^, which was 45.35–64.46%, 30.18–31.65%, and 11.10–14.28% higher than those under the N0, N1, and N2 nitrogen application levels, respectively. From the perspective of the growth stages, the promoting effect of the high-nitrogen (N3) treatment on CO_2_ emissions was mainly observed during the vigorous growth period of goji berries (mid-June to mid-August). In contrast, during the early (May) and late (after September) growth stages, the differences in CO_2_ emission flux among treatments were relatively small.

(2) N_2_O Emission Flux

**Figure 4:**
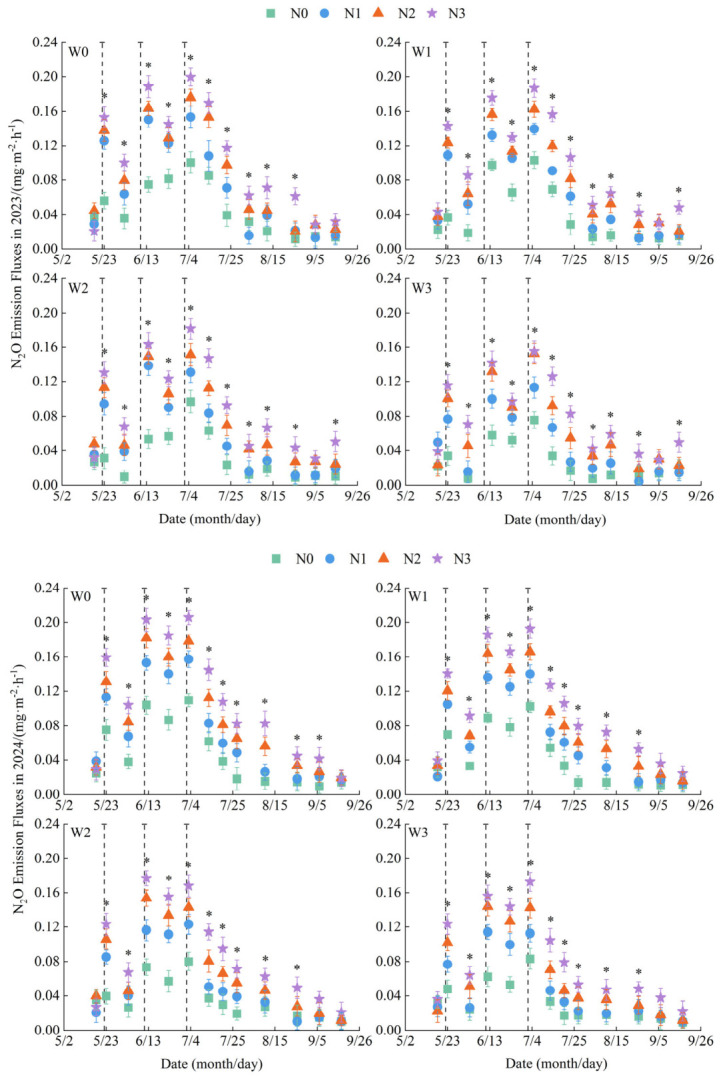
Effects of water and nitrogen regulation on N_2_O emission flux in the intercropping system of wolfberry and alfalfa in 2023~2024. * indicates extremely significant differences among nitrogen application levels under the same irrigation gradient (*p* < 0.01), and dotted lines indicate the timing of nitrogen application (along with irrigation) treatments.

The nitrogen application rate had a highly significant effect on the N_2_O emission flux in the wolfberry field (*p* < 0.01, Figure 4). During the two growing seasons, the N_2_O emission flux ranged from 0.001 to 0.20 mg·m^−2^·h^−1^ (in 2023) and from 0.001 to 0.21 mg·m^−2^·h^−1^ (in 2024). Three emission peaks occurred in May, June, and July across all treatments, with the flux increasing significantly with higher nitrogen application rates and decreasing with a greater water deficit. After August, the differences among treatments gradually diminished. Among all treatments, the W0N3 treatment exhibited the highest N_2_O emission flux, whereas the W3N0 treatment showed the lowest.

(3) CH_4_ Emission Flux

**Figure 5:**
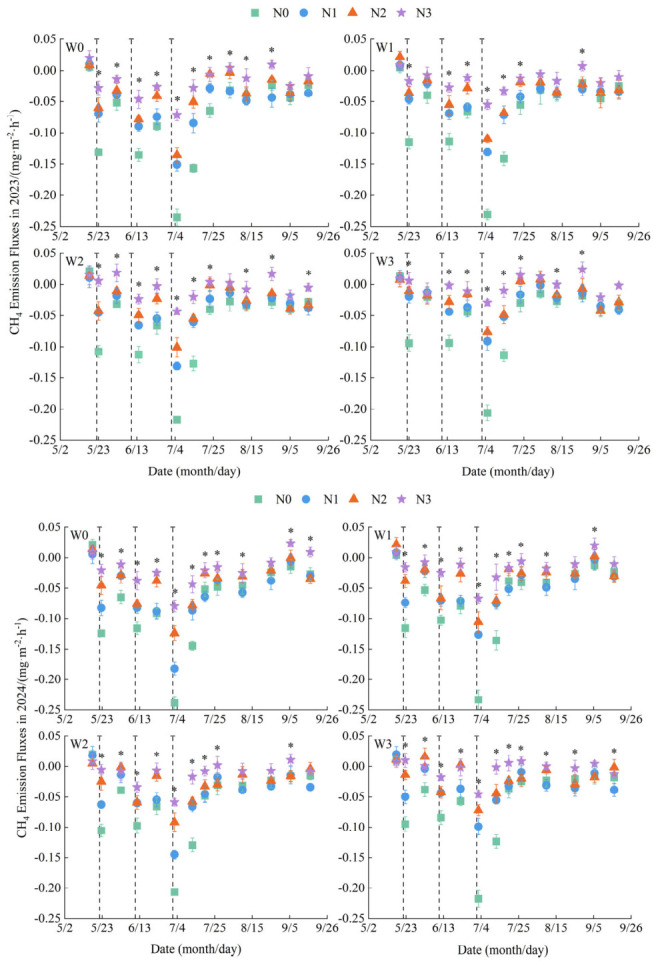
Effects of water and nitrogen regulation on CH_4_ emission flux in the intercropping system of wolfberry and alfalfa in 2023~2024. * indicates extremely significant differences among nitrogen application levels under the same irrigation gradient (*p* < 0.01), and dotted lines indicate the timing of nitrogen application (along with irrigation) treatments.

Throughout the entire growing season, the soil of the wolfberry field acted as a sink for CH_4_, exhibiting a net CH_4_ uptake (Figure 5). Over the two growing seasons, the average CH_4_ emission flux ranged from −0.24 to 0.02 mg·m^−2^·h^−1^, and showed a decreasing trend with an increasing nitrogen application rate and aggravated water deficit. The peak CH_4_ uptake across all treatments occurred between June and mid-July. Under each water gradient, the peak CH_4_ uptake in the N0 treatment was, on average, 0.05–0.18 mg·m^−2^·h^−1^ higher than that in the other nitrogen treatments. Throughout the entire growing period, the CH_4_ uptake and N_2_O emission exhibited a mirror-image pattern, with the CH_4_ uptake peak occurring after each fertilization event (accompanied by irrigation).

#### 2.2.2. Global Warming Potential

**Figure 6:**
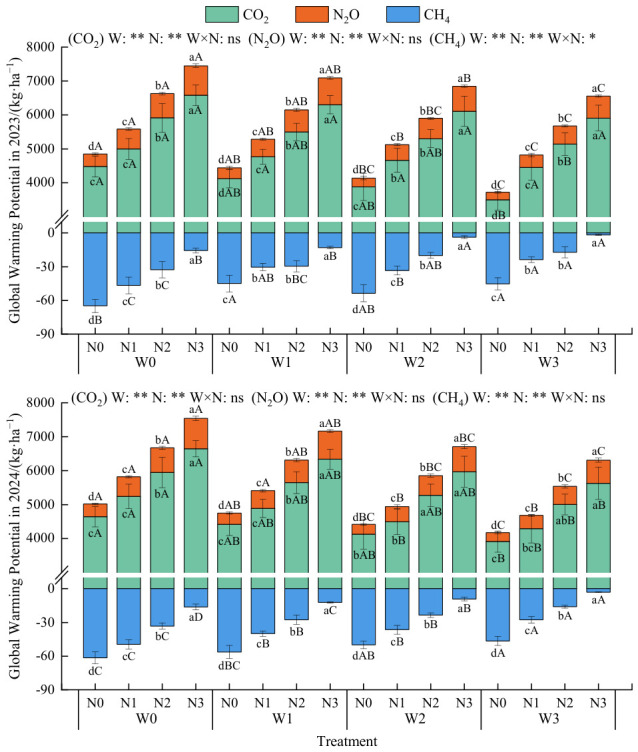
Effects of water and nitrogen regulation on the global warming potential of three soil greenhouse gases. W, N, and W × N represent the main effects of irrigation gradient, nitrogen application level, and their interaction, respectively, * and ** indicate significance at the *p* < 0.05 and *p* < 0.01 levels, respectively, and ns indicates no significance. Lowercase letters indicate differences among nitrogen application rates under the same irrigation level, and uppercase letters indicate differences among irrigation gradients under the same nitrogen application level.

**Figure 7:**
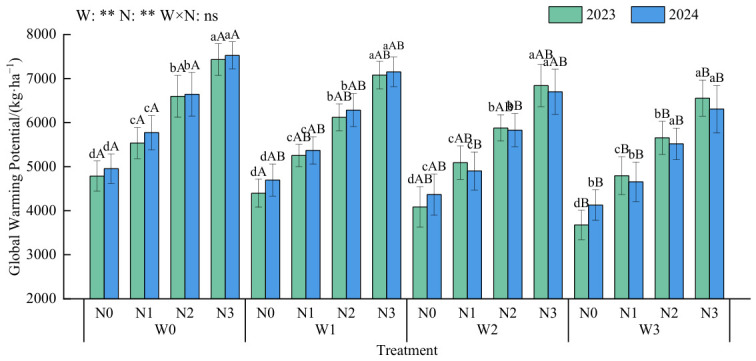
Effects of water and nitrogen regulation on the cumulative global warming potential of three soil greenhouse gases. W, N, and W × N represent the main effects of irrigation gradient, nitrogen application level, and their interaction, respectively; ** indicates significance at the *p* < 0.01 level. Lowercase letters indicate differences among nitrogen application rates under the same irrigation level, and uppercase letters indicate differences among irrigation gradients under the same nitrogen application level.

Water and nitrogen regulation significantly influenced the global warming potential of soil greenhouse gases (Table S1, Figure 6 and Figure 7, *p* < 0.01). The CO_2_ GWP accounted for 85.3–94.6% of the total GWP, making it the primary contributor to the greenhouse effect of the agricultural soil. Compared with the W0 treatment, the W3 treatment significantly reduced CO_2_ GWP and N_2_O GWP by an average of 14.55% and 31.80%, respectively, while no significant differences were observed between the W0 treatment and either the W1 or W2 treatments. Both CO_2_ GWP and N_2_O GWP exhibited an increasing trend with higher nitrogen application rates, with the N3 treatment showing average increases of 47.35% and 163%, respectively, compared with the N0 treatment. The total GWP of the N3 treatment was, on average, 49.75% higher than that of the N0 treatment. The interaction between water and nitrogen had a significant effect on N_2_O GWP and total GWP, with the highest N_2_O GWP observed in the W0N3 treatment. Compared with W0N3, the W3N3 treatment showed reductions of 25.0% in N_2_O GWP and 23.9% in total GWP, with an average reduction of 16.2% in total GWP. As the water deficit intensified, the CH_4_ uptake capacity decreased, with the CH_4_ uptake in the W3 treatment being only 43.1% and 45.4% of that in the W0 treatment. From an interannual perspective, the total GWP of all treatments was generally higher in 2024 than in 2023, with an average increase of 3.2%.

### 2.3. Correlation Analysis Between Soil Greenhouse Gas Emissions and Soil Hydrothermal Environment

**Figure 8:**
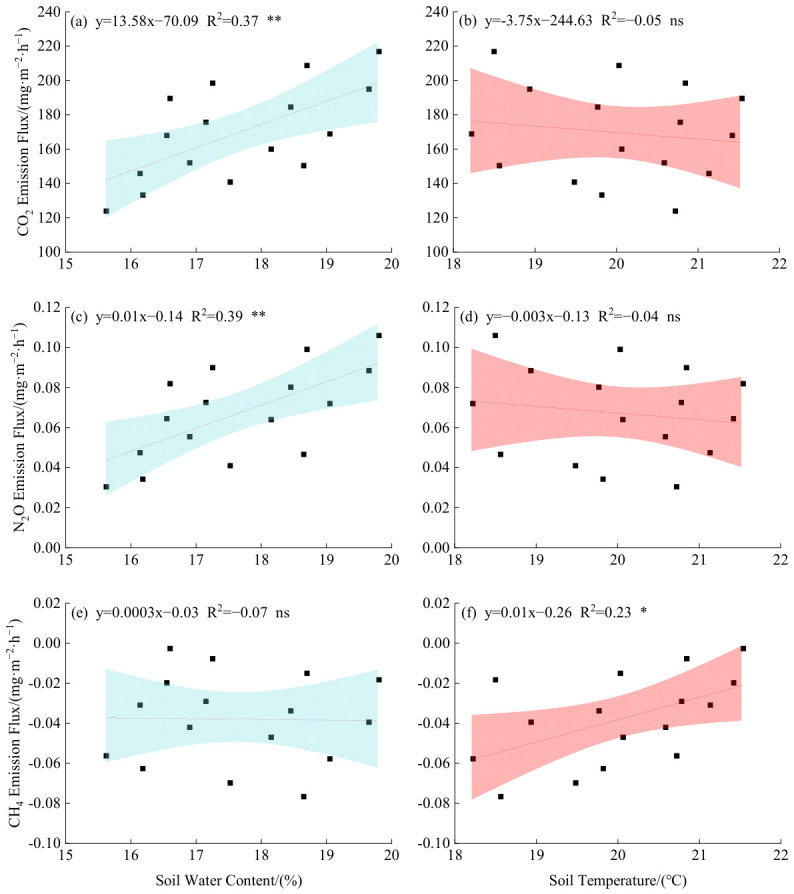
Correlation analysis between greenhouse gas emission fluxes and soil water content and soil temperature. ** indicates a significant difference (*p* < 0.01); * indicates a significant difference (*p* < 0.05); ns indicates no significant difference (*p* > 0.05). (**a**) Correlation between CO_2_ emission flux and soil water content; (**b**) Correlation between CO_2_ emission flux and soil temperature; (**c**) Correlation between N_2_O emission flux and soil water content; (**d**) Correlation between N_2_O emission flux and soil temperature; (**e**) Correlation between CH_4_ emission flux and soil water content; (**f**) Correlation between CH_4_ emission flux and soil temperature.

Correlation analysis showed the following: (Figure 8a,b) the CO_2_ emission flux was significantly positively correlated with the soil water content (*p* < 0.01), but not significantly correlated with the soil temperature (*p* > 0.05); (Figure 8c,d) the N_2_O emission flux was significantly positively correlated with the soil water content (*p* < 0.01), but not significantly correlated with the soil temperature (*p* > 0.05); and (Figure 8e,f) the CH_4_ emission flux was not significantly correlated with the soil water content (*p* > 0.05), but was significantly positively correlated with the soil temperature (*p* < 0.01).

### 2.4. The Intercropping System of Wolfberry and Alfalfa Yield and Economic Benefits

#### 2.4.1. The Intercropping System of Wolfberry and Alfalfa Yield

(1) Wolfberry yield

**Figure 9:**
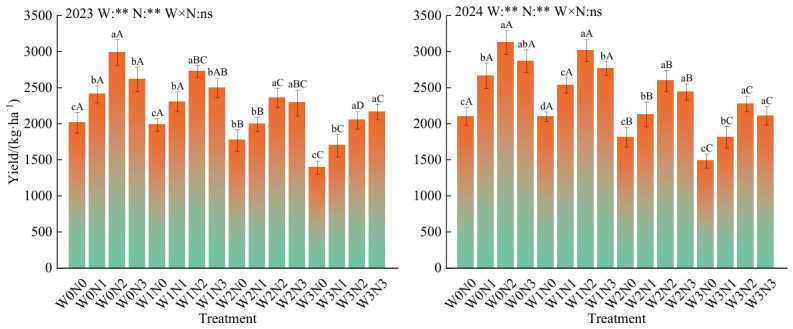
Effects of water and nitrogen regulation on wolfberry yield in the intercropping system of wolfberry and alfalfa. W, N, and W × N represent the main effect of irrigation gradient, the main effect of nitrogen application level, and their interaction effect, respectively, ** indicates significance at the *p* < 0.01 level, and ns indicates no significance. Lowercase letters indicate differences among nitrogen application rates under the same irrigation level, and uppercase letters indicate differences among irrigation gradients under the same nitrogen application level.

Irrigation and nitrogen application significantly affected the wolfberry yield (*p* < 0.01, Figure 9), but the water–nitrogen interaction had no significant effect (*p* > 0.05). At the same irrigation level, the wolfberry yield showed a trend of N2 > N3 > N1 > N0 as nitrogen application increased. Under W0 conditions, the annual average yield of the N2 treatment was significantly increased by 48.65%, 20.55%, and 11.58% compared to N0, N1, and N3, respectively; under water deficit conditions of W1, W2, and W3, the corresponding increases were 40.70%, 18.68%, and 9.07% (under W1), and 38.20%, 20.11%, and 4.74% (under W2), respectively. Under W3, the yield continued to rise in 2023 as nitrogen application increased, with the N2 treatment performing best in 2024. At the same nitrogen application level, the goji yield significantly decreased as the water deficiency worsened. Under N0 conditions, W0 increased the annual average yield of W1, W2, and W3 by 0.86%, 14.84%, and 43.01%, respectively; under N1, N2, and N3 conditions, the W0 yield increases over other water treatments were 4.90%, 23.06%, and 44.54% (under N1), 6.56%, 23.52%, and 41.46% (under N2), and 4.16%, 15.95%, and 28.28% (under N3), respectively. Over two years, W0N2 yields the highest treatment, while W3N0 yields the lowest.

(2) Alfalfa yield

**Figure 10:**
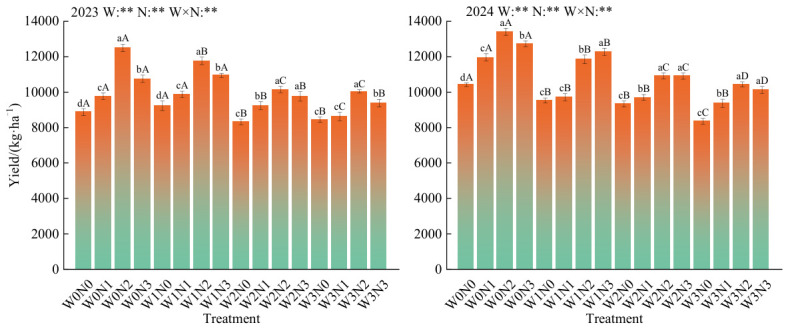
Effects of water and nitrogen regulation on alfalfa yield in the intercropping system of wolfberry and alfalfa. W, N, and W × N represent the main effects of irrigation gradient, nitrogen application level, and their interaction, respectively, and ** indicates significance at the *p* < 0.01 level. Lowercase letters indicate differences among nitrogen application rates under the same irrigation level, and uppercase letters indicate differences among irrigation gradients under the same nitrogen application level.

Irrigation, nitrogen application, and their interaction all significantly affected the alfalfa yield (*p* < 0.01, Figure 10). Under the same irrigation level, the alfalfa yield initially increased and then decreased with increasing nitrogen application. Under W0 conditions, the yield ranking was N2 > N3 > N1 > N0, with the annual average yield of the N2 treatment significantly higher than N0, N1, and N3 by 34.35%, 19.25%, and 10.38%, respectively. Under W1 conditions, there were slight inter-annual differences: in 2023, the N2 treatment had the highest yield, increasing by 27.35%, 19.11%, and 7.48% compared with N0, N1, and N3, respectively; and, in 2024, the N3 treatment was optimal, increasing by 28.84%, 26.25%, and 3.60% compared with N0, N1, and N2, respectively. Under W2 and W3 conditions, the N2 treatment had the highest yield in both years. Overall, the alfalfa yield responded less strongly to nitrogen application than wolfberry.

Under the same nitrogen application level, the alfalfa yield generally decreases as the degree of the water deficit increases. Under N0 and N1 conditions, in 2023, the yield of the W1 treatment was slightly higher than W0, and the difference between W2 and W3 treatments was not significant; and, in 2024, the trend was W0 > W1 > W2 > W3. Under N2 and N3 conditions, the yields in both years showed the same decreasing trend. Among all water–nitrogen combinations, the W0N2 treatment had the highest yield, while the W2N0 treatment had the lowest yield.

#### 2.4.2. The Intercropping System of Wolfberry and Alfalfa Economic Benefits

Irrigation and nitrogen application have a significant effect on goji berries. The total revenue, total cost, net return, and return on investment (*p* < 0.01) of the alfalfa system significantly affect the two-year return on investment and net income in 2023 (Table 1). At the same irrigation level, total benefit, net return, and return on investment all showed N2 > N3 > N1 > N0 as nitrogen application increased. Compared with N0, N1, and N3, the two-year average total returns at N2 levels increased by 40.49%, 19.74%, and 6.69%, respectively; the average net income increased by 47.51%, 23.61%, and 9.79% respectively; and the average return on investment increased by 25.77%, 16.20%, and 14.45%, respectively. The total costs increased with nitrogen application, with N3 increasing by an average of 22.40%, 41.99%, and 3.78% compared to N0, N1, and N2, respectively. At the same nitrogen application level, the total benefit, total cost, and net benefit all showed W0 > W1 > W2 > W3 as the water deficit worsened. At the W0 level, the average total return, total cost, and net return increased by 4.59%, 18.70%, 34.74%, 4.67%, 9.25%, 14.87%, 4.30%, 21.63%, and 41.53%, respectively, compared to W1, W2, and W3. The investment returns showed an upward and then declining trend as the deficit intensified, showing W1 > W0 > W2 > W3. In the two-year trial, the W0N2 treatment had the highest total and net returns, while the W1N2 treatment had the highest return on investment. W3N0 handled the lowest total returns, total costs, net returns, and return on investment.

### 2.5. Comprehensive Evaluation

#### 2.5.1. Entropy Weight–TOPSIS Method

**Figure 11:**
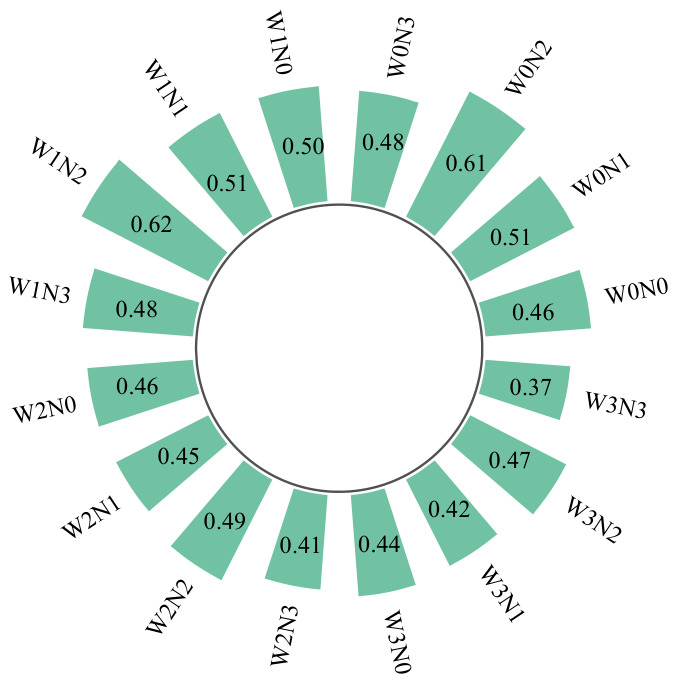
Relative closeness (comprehensive score) of each treatment based on the entropy-weighted TOPSIS method.

Based on the Entropy Weight–TOPSIS method, objective weights were assigned to each evaluation indicator (Table 2). The weights of the indicators were as follows: GWP (17.63%), alfalfa yield (21.02%), total income (14.18%), total cost (18.66%), and wolfberry yield (13.92%). On this basis, the comprehensive score of each treatment was calculated (Figure 11). The results showed that the comprehensive scores of the 16 treatments ranged from 0.37 to 0.62, with considerable variation among treatments. Among them, treatment W1N2 had the highest comprehensive score, making it the optimal treatment, while treatment W3N3 had the lowest score, making it the worst treatment.

#### 2.5.2. Integrated Water–Nitrogen Decision Model

**Figure 12:**
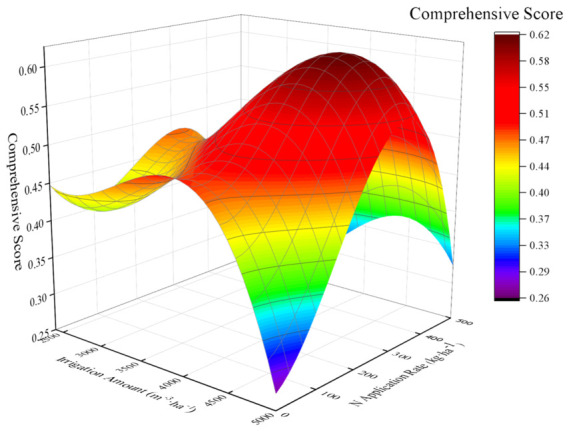
Effects of water and nitrogen regulation on comprehensive score.

The effects of the irrigation amount and nitrogen application rate on the comprehensive score followed a significant cubic curve relationship, with the following regression equation: *z* = −5.77526 × 10^−11^*x*^3^ − 1.10676 × 10^−8^*y*^3^ + 1.40732 × 10^−10^*x*^2^*y* − 7.08978 × 10^−10^*xy*^2^ + 5.61304 × 10^−7^*x*^2^ + 8.48854 × 10^−6^*y*^2^ − 5.2487 × 10^−7^*xy* − 1.75165 × 10^−3^*x* − 4.91234 × 10^−4^*y* + 2.2173. (R^2^ = 0.977, *p* < 0.01, Figure 12). The analysis of variance for the model showed that the linear and cubic terms of the irrigation amount (*x*), the linear, quadratic, and cubic terms of the nitrogen application rate (*y*), and their interaction term (*xy*) were all significant (*p* < 0.05), indicating that both the irrigation amount and nitrogen application rate had significant individual and interactive effects on the comprehensive score. The three-dimensional response surface plot exhibited a distinct convex peak shape, with the comprehensive score first increasing and then decreasing as the irrigation amount and nitrogen application rate increased. Through optimization, the optimal water–nitrogen combination range was determined as follows: an irrigation amount of 4245.16–4412.90 m^3^·ha^−1^, and a nitrogen application rate of 290.32–322.58 kg·ha^−1^.

## 3. Discussion

### 3.1. Effects of Water and Nitrogen Regulation on the Soil Hydrothermal Environment

Soil hydrothermal status is a core driving factor of energy balance and material cycling in farmland ecosystems, and its spatiotemporal dynamics directly regulate crop root water uptake, nutrient transport, and soil microbial activity. In arid and semi-arid regions, irrigation and nitrogen application, as two major regulation measures, affect the soil hydrothermal coupling characteristics of farmlands by altering the soil water redistribution and heat budget processes [9]. The results of this study show that the irrigation gradient, the nitrogen application level, and their interaction significantly affect the soil hydrothermal environment in the 0–40 cm soil layer of the intercropping of wolfberry and alfalfa. Under the same irrigation condition, the N2 treatment (300 kg·ha^−1^) had the highest soil water content, significantly higher than the N0 and N1 treatments, which is consistent with the findings of Dou et al. [10] under drip irrigation conditions. Appropriate nitrogen application can enhance the crop root biomass and root length density, thereby improving the root water uptake and retention capacity, and, consequently, increasing the soil water content in the root zone. At the same time, nitrogen improves the plant canopy structure, increases the ground cover, effectively suppresses soil evaporation, and reduces water loss [11,12]. However, under excessive nitrogen application (N3, 450 kg·ha^−1^), the soil water content did not continue to increase but showed a decreasing trend, which may be related to the increased crop transpiration water consumption and elevated soil solution osmotic pressure under high-nitrogen conditions. Under the same nitrogen application level, the soil water content significantly decreased with the increasing water deficit severity, with the W3 treatment showing a reduction of 14.80–21.10% compared to the W0 treatment, and the differences among treatments were mainly concentrated in the 0–20 cm surface soil layer, gradually diminishing with increasing depth. This phenomenon is consistent with the findings of Lei et al. [13], namely, that surface soil is affected by both irrigation and evaporation, exhibiting the largest water content variation, while the deep soil water content is relatively stable due to the buffering effect of the root water uptake. Furthermore, the differences among treatments in the 0–10 cm soil layer were more significant in 2024 than in 2023, indicating that the interannual precipitation variability affects the efficacy of water and nitrogen regulation, and, thus, the regulating effect of precipitation patterns should be fully considered when formulating irrigation schedules [14]. The soil water content was measured with a single TDR probe per plot at 30 cm from the wolfberry trunk, as in our previous study at this site [15]. We acknowledge that this single-point measurement may not fully reflect the spatial variability of the soil moisture across the intercropping system, particularly between the wolfberry row and the alfalfa strip. However, the consistent treatment effects observed across the two-year experiment suggest that our conclusions regarding treatment comparisons remain robust, and future studies could improve spatial representation by deploying multiple probes along a transect perpendicular to the crop rows.

Soil temperature is an important indicator reflecting soil thermal status, and its variation is controlled by multiple factors including solar radiation, soil water content, vegetation cover, and air temperature [16]. The results showed that the soil temperature decreased with an increasing irrigation amount. The average temperature over the entire growth period followed the order W0 (19.55 °C) < W1 (19.83 °C) < W2 (20.51 °C) < W3 (21.20 °C), with the differences being particularly significant during the full fruiting period. This is because the specific heat capacity of water is significantly higher than that of soil minerals and air; an increasing irrigation amount increases the proportion of the liquid phase in the soil and its volumetric heat capacity, resulting in a smaller temperature increase under the same solar radiation [17]. The IPCC Sixth Assessment Report noted that irrigation can reduce daytime surface temperatures by 3–8 °C by enhancing the energy consumption through transpiration and evaporation [18]. The results of this study (a reduction of approximately 2.23–2.41 °C from W0 to W3) are consistent with this trend, though the magnitude of cooling is relatively smaller, which may be related to the high vegetation coverage of the intercropping of the wolfberry and alfalfa system and the localized wetting characteristics of drip irrigation. The soil temperature decreased with an increasing nitrogen application rate. The soil temperatures in the N2 and N3 treatments were lower than those in the N1 and N0 treatments during the summer fruiting period, and the temperature peaks in both years occurred in the W3N0 treatment. This is mainly attributed to the fact that an increased nitrogen application promotes the aboveground growth of wolfberries, increasing the plant height, crown width, and leaf area index, and the dense canopy provides effective shading on the ground surface, reducing the energy flux of shortwave radiation reaching the soil surface [19]. However, under the N0 treatment, the soil temperature was relatively higher, which may be related to the restricted crop growth under nitrogen-free conditions, greater surface exposure, and direct heating of the soil by solar radiation. In addition, soil temperature in this study exhibited a typical unimodal seasonal variation, peaking in late July, which is highly coupled with regional climatic characteristics and crop phenology.

The interaction effect of water and nitrogen on both the soil water content and temperature was significant. Under a mild water deficit (W1) combined with moderate nitrogen (N2) conditions, the soil water content and temperature reached a relatively coordinated state, avoiding both excessive wetting and cooling under full irrigation and drought and heat stress under a severe water deficit. Qiu et al. [20] found that a moderate soil water deficit can improve the water use efficiency by increasing root abscisic acid synthesis, inducing moderate stomatal closure, and reducing luxury transpiration, while an appropriate nitrogen supply maintains the leaf photosynthetic capacity and ensures material production.

### 3.2. Effects of Water and Nitrogen Regulation on Soil Greenhouse Gas Emissions in the Intercropping of Wolfberry and Alfalfa

Greenhouse gas (GHG) emissions are an important ecological indicator for evaluating the sustainability of wolfberry production in arid irrigated areas [21]. The results of this study indicate that a moderate deficit irrigation combined with a medium nitrogen level is more likely to maintain the yield and economic returns while reducing the GHG emission intensity per unit yield, representing a crucial management direction for achieving a high yield, water conservation, and emission reduction in wolfberry orchards in arid regions. From the perspective of N_2_O emission mechanisms, the nitrogen application rate is a direct factor determining the emission risk, while water conditions regulate the proportion of nitrogen loss through gaseous pathways by influencing the nitrification and denitrification processes [22]. Under the N0 and N1 treatments, due to insufficient soil mineral nitrogen substrates, nitrification and denitrification are restricted, resulting in a low N_2_O emission potential. However, the inadequate nitrogen uptake by crops limits the root growth and yield formation, leading to unstable emission advantages per unit of economic return or yield [23]. Although the N3 treatment increases the supply of soil NO_3_^−^ and NH_4_^+^, when the nitrogen input exceeds the uptake capacity of wolfberry and alfalfa, the residual inorganic nitrogen becomes a substrate for N_2_O production and forms short-term emission peaks after irrigation. Previous studies have shown that the first week after irrigation and nitrogen application is a sensitive period for N_2_O and CO_2_ fluxes, and excessive water and nitrogen significantly increase GHG emissions while reducing the partial factor productivity of nitrogen [24]. Therefore, the N2 level in this study better matches the nitrogen supply with the crop demand, reducing the risks of nitrate accumulation, enhanced denitrification, and pulsed N_2_O emissions associated with high-nitrogen treatments.

The irrigation amount has a dual effect on greenhouse gas emissions. On the one hand, appropriate irrigation can alleviate drought stress, and promote the root growth of wolfberry, alfalfa cover, and soil microbial activity, thereby enhancing root respiration and organic carbon decomposition, which may increase CO_2_ emissions. On the other hand, excessive irrigation increases the soil water-filled pore space, reduces oxygen diffusion rates, and promotes the formation of localized anaerobic microsites, thereby facilitating denitrification and N_2_O production [25,26]. Although the W0 treatment favors the highest biomass production, prolonged high soil moisture following irrigation may lead to increased cumulative emissions of CO_2_ and N_2_O. In contrast, while the W2 and W3 treatments may result in lower absolute emissions due to reduced microbial activity and gas diffusion under soil water deficit, a severe deficit suppresses photosynthesis, root uptake, and alfalfa regrowth, reducing the yield carbon sinks and nitrogen uptake capacity, and, ultimately, may increase greenhouse gas emission intensity [27,28]. In the intercropping of wolfberry and alfalfa, emissions are influenced by the alfalfa cover and rhizosphere carbon inputs. Compared with bare inter-rows, the alfalfa cover increases the root exudate and litter inputs, enhancing the soil organic carbon cycling intensity and potentially increasing the soil respiration flux [29]. Under the W0N2 and W1N2 treatments, crop growth is vigorous, and the increase in CO_2_ flux likely arises from improved productivity and an enhanced rhizosphere carbon turnover. In contrast, the increase in CO_2_ under high nitrogen or excessively wet conditions may be accompanied by accelerated carbon mineralization and reduced resource use efficiency. The CH_4_ flux in upland soils and arid irrigated areas typically appears as a weak emission or net uptake, depending largely on the soil aeration, water content, and methanotroph activity. Under high-nitrogen conditions, NH_4_^+^ competes with CH_4_ for methane monooxygenase, potentially inhibiting CH_4_ oxidation and thereby reducing the soil CH_4_ uptake capacity [30]. Previous studies on legume–cereal intercropping have shown that dryland pasture systems generally exhibit a net CH_4_ uptake, with soil moisture and inorganic nitrogen being the primary factors regulating greenhouse gas fluxes [31]. Therefore, the W1N2 treatment, with non-excessive water and a moderate nitrogen supply, is more favorable for maintaining soil aeration and methanotrophic activity, whereas combinations of high water and high nitrogen, such as W0N3, may weaken the CH_4_ sink function. The correlation analysis in this study revealed significant differences in the responses of soil greenhouse gas emissions to hydrothermal factors. Emission fluxes of CO_2_ and N_2_O were significantly positively correlated with the soil water content (*p* < 0.01) but showed no significant correlation with soil temperature. This indicates that soil moisture is the key abiotic factor driving emissions of these two gases, promoting soil respiration and nitrification/denitrification processes by enhancing microbial activity and creating anaerobic microsites [32]. In contrast, the CH_4_ emission flux in this study showed no significant correlation with the soil water content but was significantly positively correlated with the soil temperature (*p* < 0.01), indicating that temperature is the dominant environmental factor regulating the soil CH_4_ uptake intensity in this system, with higher temperatures enhancing methanotroph activity.

The impact of the intercropping system of wolfberry and alfalfa on GHG emissions is also systemic. As a leguminous crop, alfalfa possesses a biological nitrogen fixation capacity, which can partially replace the external nitrogen input and reduce the soil temperature fluctuations and ineffective water evaporation through inter-row coverage, thereby helping to lower the risk of N_2_O emissions associated with a high nitrogen-dependent production [33]. However, legume fixation does not negate the need for nitrogen management; excessive nitrogen application may inhibit the nodule fixation function, shifting the system from a “biological nitrogen fixation–crop uptake” dominance to “exogenous nitrogen accumulation–gaseous loss” dominance [34]. Studies on alfalfa have indicated that controlled-release nitrogen fertilizers and appropriate mulching can reduce the GHG emission intensity and enable medium nitrogen levels to achieve a better comprehensive performance [35]. Legume–grass intercropping can also achieve a higher forage yield and lower N_2_O emissions under a low nitrogen input [26], which is consistent with the conclusions of this study. These findings demonstrate that a medium nitrogen level has a clear ecological basis, reducing the per-unit-yield emissions by enhancing the nitrogen synchrony and system uptake capacity.

### 3.3. Effects of Water and Nitrogen Regulation on Yield and Economic Benefits of the Intercropping of Wolfberry and Alfalfa

Under the same irrigation level, the wolfberry yield first increased and then decreased with an increasing nitrogen application rate, following a “diminishing returns” pattern [36]. Across all water gradients, the N2 treatment (300 kg·ha^−1^) achieved the highest yield. This pattern is closely related to the regulatory effect of nitrogen on carbon allocation and source–sink relationships in wolfberry: appropriate nitrogen application meets the nutrient demand during the reproductive growth stage, promoting flower bud differentiation and fruit sink establishment; however, excessive nitrogen application (450 kg·ha^−1^) tends to induce excessive vegetative growth, significantly increasing the canopy closure, deteriorating ventilation and light conditions, inhibiting the allocation of photosynthetic products to reproductive organs, and, ultimately, leading to a yield reduction [37]. Under the same nitrogen level, the wolfberry yield decreased significantly with an increasing water deficit severity (*p* < 0.01), indicating that water availability is the dominant limiting factor for the wolfberry yield in this region. A water deficit reduces the yield through multiple pathways: on the one hand, it inhibits photosynthesis by reducing the leaf stomatal conductance; on the other hand, it reduces the root uptake of nitrogen and other nutrients and their transport to aboveground parts, while also exacerbating the damage of high-temperature stress to the cell membrane system, collectively leading to a yield reduction [38]. Adequate irrigation maintains a favorable plant water and nutrient status, ensuring the normal functioning of key physiological processes during the reproductive growth period [39].

Under the same nitrogen level, the alfalfa yield generally decreased with an increasing water deficit severity, though interannual differences were observed. In 2023, under the N0 and N1 conditions, the W1 treatment (mild deficit) achieved a higher yield than the W0 treatment, exhibiting a compensatory effect [40]. This may be related to the well-developed root system of alfalfa: a moderate water stress can induce deeper root growth, improving the water use efficiency from deeper soil layers [41]. Furthermore, as a legume crop, alfalfa’s nodule nitrogen fixation activity may be temporarily upregulated under a mild water deficit, partially compensating for the reduction in exogenous nitrogen supply and further promoting the compensatory effect [42]. However, in 2024, the W0 treatment achieved the highest yield under all nitrogen levels, and no compensatory effect was observed, indicating that the compensatory effect of a water deficit is strongly dependent on the interannual climatic variability. The 2023 growing season likely had more favorable rainfall distribution or lower potential evapotranspiration conditions, providing an environmental basis for the occurrence of the compensatory effect.

The total revenue, net revenue, and return on investment (ROI) were all highly significantly affected by water and nitrogen regulation, and the interaction effect of water and nitrogen on ROI was also highly significant, indicating a significant synergistic amplification effect of water–nitrogen coupling on economic benefits. The patterns of total revenue and net revenue were highly consistent with the yields of wolfberry and alfalfa, suggesting that crop yield is the core factor determining total revenue. ROI was generally optimal under the N2 treatment across all treatments, indicating that moderate nitrogen application has a comprehensive advantage in terms of capital use efficiency [43]. The total cost increased with an increasing nitrogen application rate and irrigation amount. The W0N2 treatment achieved the highest total revenue, net revenue, and ROI in both years, indicating that adequate irrigation combined with moderate nitrogen application is the optimal combination for maximizing economic benefits. In 2024, the ROI of the W1N2 treatment was slightly higher than that of the W0N2 treatment, suggesting that a moderate water deficit did not disrupt the complementary structure of the system but rather achieved a synergy between economic benefits and resource use efficiency by reducing irrigation costs, maintaining moderate to high yields, and avoiding inefficient high-nitrogen inputs.

## 4. Materials and Methods

### 4.1. Description of the Experimental Site

**Figure 13:**
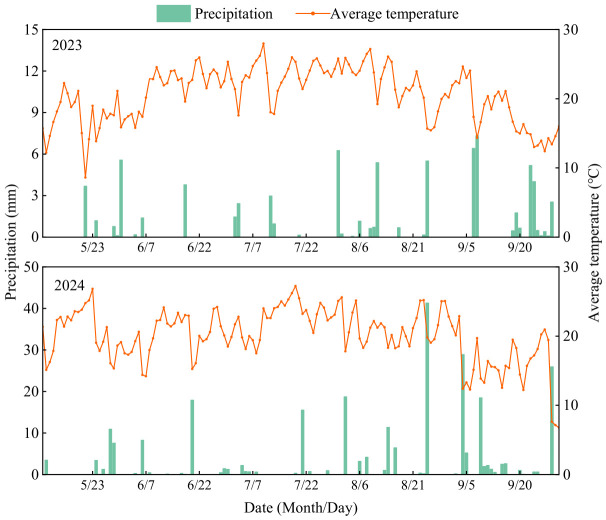
Daily precipitation and average air temperature during the fertility period of wolfberry in the experimental site.

The experiment was conducted from May to September in 2023 and 2024 at the Irrigation Experiment Station of the Jingtaichuan Electric Power Irrigation Water Resources Utilization Center in Gansu Province (37°12′ N, 104°5′ E; mean altitude 1562 m). The experimental area is located in the middle and upper reaches of the Yellow River and has a temperate arid continental climate, with multi-year average values of 2652 h of sunshine duration, 6.18 × 10^5^ J·cm^−2^ of solar radiation, 8.6 °C of air temperature, 191.6 mm of precipitation, 2761 mm of evaporation, and 191 frost-free days. The soil is sandy loam. According to the IUSS Working Group WRB in 2022, the soil is classified as Calcaric Fluvisol (Loamic). According to the USDA Soil Taxonomy, it is classified as Typic Haplocambids. Its basic physicochemical properties are shown in Table 3. During the experimental periods in 2023 and 2024, the daily mean air temperatures were 20.61 °C and 20.06 °C, respectively, and the total effective precipitation amounts were 112.47 mm and 259.02 mm, respectively (Figure 13).

### 4.2. Experimental Design

The tested wolfberry variety was ‘Ningqi No. 5’, and the tested alfalfa variety was ‘Longdong Alfalfa’. The experiment used a completely randomized block design with two factors: irrigation gradient and nitrogen application level (Table 4). The irrigation gradients were set as full irrigation (75–85% θ_FC_, W0), mild water deficit (65–75% θ_FC_, W1), moderate water deficit (55–65% θ_FC_, W2), and severe water deficit (45–55% θ_FC_, W3). The nitrogen application levels were set as no nitrogen (0, N0), low nitrogen (150 kg·ha^−1^, N1), medium nitrogen (300 kg·ha^−1^, N2), and high nitrogen (450 kg·ha^−1^, N3). A total of 16 treatments were established, each with three replications. The plot area was 76.5 m^2^ (10.2 m × 7.5 m), with a 1 m wide buffer strip between adjacent plots. Fertilizer was applied using a Venturi injector(Shandong Muyang Water Conservation Irrigation Co., Ltd., Jinan, China). Nitrogen fertilizer (urea, 46% N) was applied in three split applications at a ratio of 6:2:2 (in 2023: 22 May, 10 June, and 2 July; in 2024: 22 May, 11 June, and 2 July). Phosphorus fertilizer (superphosphate, 12% P_2_O_5_, 130 kg·ha^−1^) and potassium fertilizer (potassium chloride, 60% K_2_O, 130 kg·ha^−1^) were applied as basal fertilizers in a single application on May 6, 2023 and May 6, 2024, respectively. Field agronomic management practices such as weeding and spraying were consistent with local production practices.

**Figure 14:**
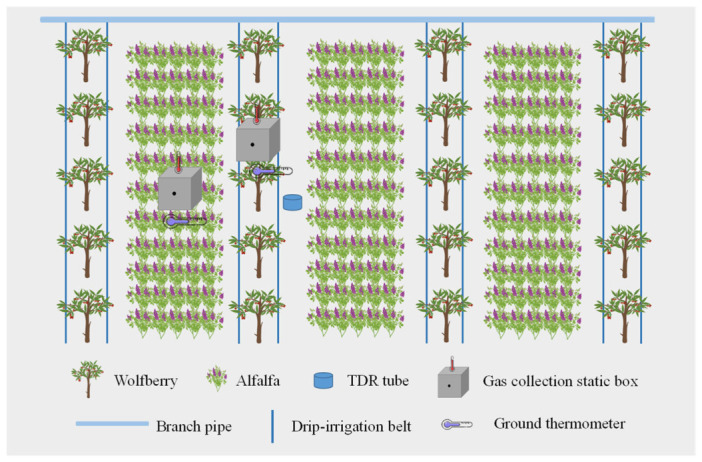
Plot layout of the test site.

The tested wolfberry plants were two-year-old seedlings transplanted on 12 April 2021, with a plant spacing of 1.5 m and a row spacing of 3 m. The wolfberry growing season was divided into four key stages: vegetative growth period (late April to mid-June), full flowering period (late June to early July), full fruiting period (mid-July to mid-August), and autumn fruiting period (late August to mid-September). Alfalfa was drill-seeded between the wolfberry rows at a seeding rate of 13 kg·ha^−1^, starting 0.9 m from the wolfberry trunk, with a row spacing of 0.3 m (Figure 14). Alfalfa was cut on 18 June, 22 July, and 16 September in 2023, and on 16 June, 25 July, and 19 September in 2024. Drip irrigation was adopted, with drip tape spacing of 0.3 m, a designed emitter flow rate of 2 L·h^−1^, and an emitter spacing of 0.3 m.

### 4.3. Determination and Calculation of Indicators

#### 4.3.1. Meteorological Data

Meteorological data such as precipitation and air temperature during the experimental period were obtained using a WSTQ Tianqi small agricultural weather station.

#### 4.3.2. Soil Hydrothermal Properties

(1) Soil water content

In each experimental plot, a 150 cm-long time-domain reflectometry (TDR) probe tube was randomly installed at 30 cm from the center of the wolfberry trunk [15]. Soil volumetric water content in the 0–40 cm soil layer was measured every 5 days using a PICO-BT TDR instrument (IMKO, Pfullendorf, BW, Germany). Additional measurements were taken before and after each irrigation event and after rainfall events, and the TDR measurements were periodically calibrated using the oven-drying method. The obtained soil water content data were used to determine whether the soil water content in each plot reached the designed lower limit of irrigation, and the actual irrigation amount was calculated accordingly.

(2) Soil temperature

In the center of each plot, approximately 10 cm from the static chamber, soil temperature in the 5 cm soil layer was monitored using a soil thermometer (right-angle geothermometer). This depth is most relevant to surface GHG emissions. Soil temperature monitoring was conducted synchronously with greenhouse gas measurements.

#### 4.3.3. Greenhouse Gases

Greenhouse gases were collected using the closed static chamber method [44]. The static chamber had dimensions of 60 × 60 × 60 cm, and the base had dimensions of 60 × 60 × 15 cm, inserted 10 cm into the soil. During gas sampling, the static chamber was placed on the water trough of the base, and water was added to create a seal. A thermometer was inserted into the central port at the top of the static chamber. A gas sampling port and a fan power cord port were installed on the left side of the chamber, and two 12 V small fans (powered by an external battery) were installed inside the chamber to mix the gas. During non-sampling periods, the static chamber was removed to ensure that light, air temperature, soil temperature, and humidity inside and outside the chamber remained consistent. Greenhouse gas sampling was conducted between 9:00 and 11:00 a.m. after each fertilization, irrigation, or precipitation event. After the static chamber was set up, it was allowed to stand for 3 min, after which 40 mL of gas was extracted from the gas sampling port using a 50 mL syringe, followed by extractions every 10 min for a total of four extractions. The collected gas samples were analyzed for CO_2_, N_2_O, and CH_4_ concentrations using a gas chromatograph (Shimadzu GC-2010 Pro, Shimadzu Corporation, Kyoto, Japan) [45].

(1) Flux, *F*, mg·m^−2^·h^−1^

(1) $$F=\rho \cdot H\cdot \frac{dc}{dt}\cdot \frac{273}{273+T}$$

where *ρ* is the density of CO_2_, N_2_O, or CH_4_ under standard conditions (kg·m^−3^); *H* is the effective height of the static chamber (m); *dc*/*dt* is the gas concentration difference (mL·m^−3^·h^−1^); and *T* is the average temperature inside the static chamber (°C).

(2) Cumulative Emissions, *CE*, mg·m^−2^

(2) $$CE=\sum_{i=1}^{n}(\frac{F_{i}+F_{i+1}}{2})\times (t_{i+1}-t_{i})\times 24$$

*F_i_*—emission flux of CO_2_, N_2_O, or CH_4_ at the *i*-th sampling, mg·m^−2^·h^−1^; *F_i_*_+1_—emission flux of CO_2_, N_2_O, or CH_4_ at the (*i* + 1)-th sampling, mg·m^−2^·h^−1^; *t_i_*—time of the *i*-th sampling, d; and *t_i_*_+1_—time of the (*i* + 1)-th sampling, d.

(3) Global Warming Potential, *GWP*, kg·ha^−1^

(3) $$GWP=CE({\mathrm{CO}}_{2})+CE(N_{2}O)\;\times \;273+CE({\mathrm{CH}}_{4})\;\times \;27$$

*CE*(CO_2_), *CE*(N_2_O), and *CE*(CH_4_) represent the cumulative emissions of CO_2_, N_2_O, and CH_4_, respectively, mg·m^−2^. According to statistics [18], the global warming potential values per unit mass of N_2_O and CH_4_ over a 100-year horizon are 273 and 27 times that of CO_2_.

#### 4.3.4. Yield

Starting from the full fruiting period of wolfberry, the fruits were picked, dewaxed, and dried at intervals of 7 days on a per-plant basis. The yield per unit area (Y, kg·ha^−1^) was calculated based on the plot area. For the intercropping treatment, a representative 1 m^2^ alfalfa plot was selected. Alfalfa was cut when 50% of the plants were flowering, leaving a stubble height of 5 cm each time. The fresh weight was measured immediately in the field, then the samples were oven-dried at 105 °C for 30 min to deactivate enzymes, followed by drying at 65 °C to constant weight, and the alfalfa yield was calculated.

#### 4.3.5. Economic Benefits

Based on the yields of wolfberry and alfalfa and their market prices, the economic benefits were analyzed.

The expenditures mainly included the following: urea at 3.25 yuan·kg^−1^, superphosphate at 1 yuan·kg^−1^, potassium chloride at 4 yuan·kg^−1^, irrigation electricity and water fees at 0.33 yuan·m^−3^; labor cost at 150 yuan·person^−1^·day^−1^, and wolfberry picking fee at 3.5 yuan·kg^−1^. Other expenses for each treatment were calculated according to actual conditions. The average price of wolfberry was 36 yuan·kg^−1^, and the average price of alfalfa was 2.1 yuan·kg^−1^.

(1) Total Revenue, TR, yuan·ha^−1^

(4) $$TR=(Y_{g}\;\times \;P_{g})+(Y_{a}\;\times \;P_{a})$$

where *Y_g_* and *Y_a_* are the yields of wolfberry and alfalfa (kg·ha^−1^), respectively; and *P_g_* and *P_a_* are the market prices of wolfberry and alfalfa (yuan·kg^−1^), respectively.

(2) Total Cost, TC, yuan·ha^−1^

(5) $$TC=C_{f}\;+C_{i}\;+C_{l}+C_{o}$$

where *C_f_* is the fertilizer cost (urea, superphosphate, and potassium chloride); *C_i_* is the irrigation electricity and water fee; *C_l_* is the total labor cost (including the specific wolfberry picking fee); and *C_o_* represents other operating expenses calculated according to actual conditions.

(3) Net Revenue, NR, yuan·ha^−1^

(6) NR =TR−TC

(4) Return on Investment, ROI

(7) ROI =NR/TC

#### 4.3.6. Entropy Weight–TOPSIS Model

(1) Determining the weights of indicators using the entropy weight method [46]

Given m evaluation objects and n evaluation indicators, the values of each evaluation indicator *Y_ij_* (*i* = 1, 2, 3, …, *m*; *j* = 1, 2, 3, …, *n*) are normalized to obtain the normalized indicator values *X_ij_*, and then the weights *W_ij_* of each indicator are calculated:

(8) $$P_{ij}\;=\frac{X_{ij}}{\sum_{i=1}^{m}X_{ij}}$$

(9) $$e_{j}=-[\ln\;m{]}^{-1}\times \sum_{i=1}^{m}\;P_{ij}\times \ln\;P_{ij}$$

(10) $$d_{j}=1-e_{j}$$

(11) $$W_{j}=\frac{1-e_{j}}{\sum_{j=1}^{n}\left(1-e_{j}\right)}$$

In the formula, *P_ij_* is the contribution degree of the i-th evaluation object for the j-th indicator; *e_j_* is the information entropy value; *d_j_* is the information utility value; and *W_j_* is the weight obtained for each indicator (%).

(2) Comprehensive evaluation using the TOPSIS method

(1) To objectively evaluate each treatment, the TOPSIS method was used to construct the weighted normalization matrix.

(12) $$R_{ij}=W_{j}\times X_{ij}$$

(2) The positive ideal solution *R^+^* and negative ideal solution *R*^−^ were determined: the closer the value of the indicator vector is to the positive ideal solution, the better the performance is; conversely, the closer it is to the negative ideal solution, the worse the performance is.

(13) $$R^{+}=max(R_{i1},R_{i2},\ldots ,R_{im})$$

(14) $$R^{-}=min(R_{i1},R_{i2},\ldots ,R_{im})$$

(3) Optimal solution distance *D^+^* and worst solution distance *D^−^*

(15) $$D^{+}=\sqrt{\sum_{j=1}^{n}(R_{ij}-R_{j^{+}}{)}^{2}}$$

(16) $$D^{-}=\sqrt{\sum_{j=1}^{n}(R_{ij}-R_{j^{-}}{)}^{2}}$$

(4) Relative Closeness *Ci*

(17) $$C_{i}=\frac{D_{i}^{-}}{D_{i}^{+}+D_{i}^{-}}$$

### 4.4. Data Analysis

Microsoft Excel 2019 was used for data organization. One-way analysis of variance (One-way ANOVA) and Duncan’s method in IBM SPSS Statistics 26.0 software were used for variance analysis and multiple comparisons, and two-way ANOVA was used to analyze the effects of irrigation gradient, nitrogen application level, and their interaction, with a significance level of *p* < 0.05. Design-Expert 13.0 software was used for response surface regression analysis to establish the regression equation of irrigation amount and nitrogen application rate on the comprehensive score. Origin 2021 software was used for graphing.

## 5. Conclusions

Water and nitrogen regulation significantly affects the soil hydrothermal environment, greenhouse gas emissions, crop yield, and economic benefits of the intercropping system of wolfberry and alfalfa. The soil water content increases with higher nitrogen application rates but decreases with a more severe water deficit, whereas the soil temperature shows the opposite trend. The emission fluxes of CO_2_ and N_2_O increase with higher nitrogen application rates but decrease with a more severe water deficit. CH_4_ acts as a sink, with its uptake decreasing as nitrogen application increases and the water deficit intensifies. CO_2_ is the dominant contributor to the global warming potential of the intercropping system of wolfberry and alfalfa, accounting for 85.3–94.6% of the total. The emission fluxes of CO_2_ and N_2_O are significantly positively correlated with the soil water content, while the CH_4_ emission flux is significantly positively correlated with the soil temperature. Notably, an excessive nitrogen application (N3) substantially increased the total GWP by 49.75% compared to N0. The W0N2 treatment achieves the highest system yield and net profit, whereas the W1N2 treatment exhibits the highest return on investment. Based on the comprehensive evaluation using the entropy weight–TOPSIS method and response surface optimization, an irrigation amount of 4245–4413 m^3^·ha^−1^ coupled with a nitrogen application rate of 290–323 kg·ha^−1^ can effectively coordinate the production benefits and environmental effects, thereby achieving a synergistic development of high yield, high efficiency, and emission reduction in the intercropping system of wolfberry and alfalfa.

## Figures and Tables

**Table 1:** Effects of water and nitrogen regulation on farmland economic benefits in the intercropping system of wolfberry and alfalfa.

Year	Treatment	Total Revenue (×10^4^ yuan·ha^−1^)	Total Cost (×10^4^ yuan·ha^−1^)	Net Revenue (×10^4^ yuan·ha^−1^)	Return on Investment
2023	W0N0	9.11 ± 0.47 cA	2.21 ± 0.08 cA	6.90 ± 0.45 cA	3.12 ± 0.11 bB
	W0N1	10.71 ± 0.62 bA	2.51 ± 0.05 bA	8.21 ± 0.62 bA	3.27 ± 0.06 bB
	W0N2	13.37 ± 0.35 aA	2.62 ± 0.10 abA	10.75 ± 0.33 aA	4.10 ± 0.13 aA
	W0N3	11.66 ± 0.78 bA	2.71 ± 0.06 aA	8.95 ± 0.58 bA	3.3 ± 0.04 bA
	W1N0	9.08 ± 0.51 cA	2.04 ± 0.09 dB	7.04 ± 0.27 cA	3.45 ± 0.09 cA
	W1N1	10.37 ± 0.69 bA	2.24 ± 0.04 cB	8.13 ± 0.49 bA	3.63 ± 0.12 bA
	W1N2	12.27 ± 0.43 aB	2.43 ± 0.07 bB	9.84 ± 0.64 aB	4.05 ± 0.05 aA
	W1N3	11.28 ± 0.74 abAB	2.66 ± 0.11 aA	8.62 ± 0.38 bAB	3.24 ± 0.08 dAB
	W2N0	8.11 ± 0.58 bA	2.05 ± 0.05 bB	6.06 ± 0.53 cB	2.95 ± 0.14 cB
	W2N1	9.11 ± 0.36 bB	2.17 ± 0.08 bB	6.94 ± 0.29 bcB	3.19 ± 0.07 bB
	W2N2	10.61 ± 0.65 aC	2.39 ± 0.06 aB	8.22 ± 0.46 aC	3.44 ± 0.10 aB
	W2N3	10.28 ± 0.49 aBC	2.47 ± 0.10 aB	7.81 ± 0.67 abB	3.16 ± 0.04 bB
	W3N0	6.78 ± 0.72 cB	1.98 ± 0.04 cB	4.80 ± 0.35 cC	2.42 ± 0.12 bC
	W3N1	7.91 ± 0.54 bC	2.23 ± 0.09 bB	5.68 ± 0.51 bC	2.55 ± 0.06 bC
	W3N2	9.48 ± 0.38 aD	2.35 ± 0.07 abB	7.13 ± 0.24 aD	3.03 ± 0.13 aC
	W3N3	9.75 ± 0.67 aC	2.43 ± 0.05 aB	7.32 ± 0.42 aC	3.01 ± 0.08 aC
	ANOVA				
	W	**	**	**	**
	N	**	**	**	**
	W × N	ns	ns	*	**
2024	W0N0	9.74 ± 0.46 cA	2.32 ± 0.08 cA	7.42 ± 0.59 cA	3.19 ± 0.05 cB
	W0N1	12.10 ± 0.71 bA	2.54 ± 0.06 bA	9.56 ± 0.31 bA	3.76 ± 0.11 bA
	W0N2	14.07 ± 0.53 aA	2.65 ± 0.10 abA	11.42 ± 0.55 aA	4.31 ± 0.09 aA
	W0N3	12.99 ± 0.39 bA	2.76 ± 0.04 aA	10.23 ± 0.37 bA	3.70 ± 0.14 bA
	W1N0	9.54 ± 0.76 cAB	2.19 ± 0.09 cAB	7.35 ± 0.68 dA	3.35 ± 0.07 cA
	W1N1	11.14 ± 0.41 bA	2.42 ± 0.07 bBC	8.72 ± 0.43 cA	3.60 ± 0.10 bA
	W1N2	13.44 ± 0.64 aA	2.58 ± 0.05 bAB	10.86 ± 0.26 aA	4.38 ± 0.04 aA
	W1N3	12.52 ± 0.57 aA	2.67 ± 0.08 aA	9.85 ± 0.61 bA	3.69 ± 0.12 bA
	W2N0	8.48 ± 0.33 cB	2.07 ± 0.11 bBC	6.41 ± 0.34 cA	3.10 ± 0.06 bB
	W2N1	9.70 ± 0.68 bB	2.39 ± 0.06 aB	7.31 ± 0.48 bB	3.05 ± 0.13 bB
	W2N2	11.62 ± 0.44 aB	2.55 ± 0.04 aAB	9.07 ± 0.22 aB	3.55 ± 0.08 aB
	W2N3	11.07 ± 0.59 aB	2.51 ± 0.10 aB	8.56 ± 0.56 aB	3.41 ± 0.05 aB
	W3N0	7.09 ± 0.73 cC	1.97 ± 0.07 bC	5.12 ± 0.39 cB	2.60 ± 0.11 cC
	W3N1	8.49 ± 0.48 bC	2.06 ± 0.05 bC	6.43 ± 0.65 bB	3.12 ± 0.09 bB
	W3N2	10.37 ± 0.55 aC	2.28 ± 0.09 aB	8.09 ± 0.28 aC	3.55 ± 0.14 aB
	W3N3	9.71 ± 0.37 aC	2.39 ± 0.08 aB	7.32 ± 0.52 abC	3.06 ± 0.07 bC
	ANOVA				
	W	**	**	**	**
	N	**	**	**	**
	W × N	ns	ns	ns	**

Note: W, N, and W × N represent the main effects of irrigation gradient, nitrogen application level, and their interaction, respectively, ** indicates significance at the *p* < 0.01 level, * indicates significance at the *p* < 0.05 level and ns indicates no significance. Lowercase letters indicate differences among nitrogen application rates under the same irrigation level, and uppercase letters indicate differences among irrigation gradients under the same nitrogen application level.

**Table 2:** Weights of each indicator calculated based on the TOPSIS method.

Indicator	GWP	Wolfberry Yield	Alfalfa Yield	Total Revenue	Total Cost
Information entropy value	0.93	0.95	0.92	0.94	0.93
Information utility value	0.07	0.05	0.08	0.06	0.07
Weight (%)	17.63	13.92	21.02	14.18	18.66

**Table 3:** Basic physical and chemical properties of soil.

Bulk Density (g·cm^−3^)	Organic Matter Content (g·kg^−1^)	Total N Content (g·kg^−1^)	Total P Content (g·kg^−1^)	Total K Content (g·kg^−1^)	Available N Content (mg·kg^−1^)	Available P Content (mg·kg^−1^)	Available K Content (mg·kg^−1^)	Field Capacity (%)	pH
1.63	13.20	1.62	1.32	34.03	74.51	26.31	173	24.1%	8.11

Note: Soil samples were collected from the Ap horizon (0–20 cm depth).

**Table 4:** Experimental design.

Treatment	Irrigation Gradient	Irrigation Amount (m^−3^·ha^−1^)		Total N Application Rate (kg·ha^−1^)	Nitrogen Application Distribution in 2023 (kg·ha^−1^)			Nitrogen Application Distribution in 2024 (kg·ha^−1^)		
		2023	2024		5/22	6/10	7/2	5/22	6/11	7/2
W0N0	Full irrigation 75%~85% θ_FC_	4526.15	4228.34	0	0	0	0	0	0	0
W0N1		4173.84	4665.71	150	90	30	30	90	30	30
W0N2		4639.06	4726.89	300	180	60	60	180	60	60
W0N3		4764.73	4853.42	450	270	90	90	270	90	90
W1N0	Mild water deficit 65%~75% θ_FC_	3847.02	3956.18	0	0	0	0	0	0	0
W1N1		3548.71	3652.45	150	90	30	30	90	30	30
W1N2		3943.93	4058.63	300	180	60	60	180	60	60
W1N3		4051.6	4169.27	450	270	90	90	270	90	90
W2N0	Moderate water deficit 55%~65% θ_FC_	3167.89	3273.91	0	0	0	0	0	0	0
W2N1		2923.58	2900.25	150	90	30	30	90	30	30
W2N2		3248.80	3226.02	300	180	60	60	180	60	60
W2N3		3338.47	3315.69	450	270	90	90	270	90	90
W3N0	Severe water deficit 45%~55% θ_FC_	2488.76	2465.43	0	0	0	0	0	0	0
W3N1		2423.45	2400.12	150	90	30	30	90	30	30
W3N2		2553.67	2530.89	300	180	60	60	180	60	60
W3N3		2625.34	2602.56	450	270	90	90	270	90	90

## Data Availability

All data are incorporated into the article.

## Supplementary Materials

The following supporting information can be downloaded at: https://www.mdpi.com/article/10.3390/plants15132038/s1, Table S1: Effects of water and nitrogen regulation on cumulative greenhouse gas emissions and global warming potential in soil.
