# Supplementary material for: Optimizing Irrigation and Nitrogen Inputs for Balancing Greenhouse Gas Mitigation, Productivity, and Profitability in an Intercropping System of Wolfberry and Alfalfa
Source: Plants (Basel). 2026 Jul 1;15(13):2038. doi: 10.3390/plants15132038 (PMC13363920; doi:10.3390/plants15132038)
Supplement: Supplementary file 1 [file plants-15-02038-s001.zip › plants-4385688-supplementary.pdf]

Effects of water and nitrogen regulation on cumulative greenhouse gas emissions and global warming potential in soil

Year	Treatment	CE(CO_2) ($g·m^{-2}$)	CE(N_2O) ($mg·m^{-2}$)	CE(CH_4) ($mg·m^{-2}$)	GWP ($kg·ha^{-1}$)	GHGI (kg CO_2−eq·kg^{-1})
2023	W0N0	448.02±30.78cA	136.09±10.32dA	−240.30±21.54dB	4786.84±341.78dA	2.38±0.12bAB
	W0N1	499.64±31.03cA	215.14±13.48cA	−172.95±27.25cC	5537.03±354.45cA	2.30±0.05bC
	W0N2	591.51±42.38bA	261.57±15.86bA	−120.95±26.89bC	6596.52±474.35bA	2.21±0.18bB
	W0N3	658.05±30.12aA	317.29±20.13aA	−57.87±8.14aB	7431.07±358.35aA	2.84±0.08aB
	W1N0	412.10±26.66dAB	118.29±15.38dAB	−166.39±27.45cA	4399.00±315.99dA	2.22±0.14bB
	W1N1	476.77±22.03cA	189.58±10.93cAB	−112.32±12.24bAB	5254.92±253.44cAB	2.28±0.03bC
	W1N2	549.89±25.54bAB	238.06±16.46bAB	−109.66±18.47bBC	6119.19±305.32bAB	2.25±0.16bB
	W1N3	630.08±27.04aA	289.46±15.22aAB	−48.14±4.30aB	7078.02±313.11aAB	2.83±0.10aB
	W2N0	388.03±40.18cAB	95.34±18.30dBC	−198.43±28.04dAB	4087.00±459.32dAB	2.31±0.14cB
	W2N1	466.32±34.88bA	168.81±10.41cB	−123.87±14.76cB	5090.60±381.20cAB	2.55±0.06bB
	W2N2	530.06±26.74bAB	218.87±10.51bBC	−74.01±9.72bAB	5878.13±298.71bAB	2.49±0.13bcAB
	W2N3	610.87±44.54aA	270.38±12.77aB	−13.68±4.36aA	6843.14±481.43aAB	2.99±0.04aA
	W3N0	349.85±30.24dB	81.56±9.59dC	−167.45±19.86cA	3675.93±333.94dB	2.64±0.17bA
	W3N1	445.53±37.43cA	133.52±19.33cC	−87.86±9.08bA	4796.08±429.52cB	2.83±0.09abA
	W3N2	513.96±34.01bB	194.76±11.58bC	−63.38±18.22bA	5654.18±376.63bB	2.76±0.15bA
	W3N3	590.71±37.66aA	237.81±12.63aC	−5.98±2.12aA	6554.70±411.65aB	3.03±0.07aA
	ANOVA					
	W	**	**	**	**	**
	N	**	**	**	**	**
	W×N	ns	ns	*	ns	ns
2024	W0N0	464.49±30.24cA	136.50±9.38dA	−227.52±19.46dC	4956.11±333.26dA	2.36±0.11bB
	W0N1	524.20±35.77cA	212.39±10.84cA	−183.59±15.63cC	5772.25±391.51cA	2.17±0.18bB
	W0N2	594.68±44.72bA	267.23±16.05bA	−123.05±10.30bC	6643.11±493.79bA	2.12±0.05bB
	W0N3	664.67±24.21aA	327.98±23.32aA	−60.10±9.33aD	7525.85±308.28aA	2.63±0.14aB
	W1N0	441.76±32.63cAB	122.79±11.99dAB	−208.55±21.39dBC	4696.50±364.80dAB	2.24±0.08bB
	W1N1	488.77±26.76cAB	191.18±14.55cA	−148.07±9.18cB	5369.64±309.80cAB	2.12±0.16bB
	W1N2	564.95±32.22bAB	243.10±17.28bAB	−102.24±14.96bB	6285.55±373.41bAB	2.08±0.03bB
	W1N3	633.59±29.46aAB	303.73±15.10aAB	−45.28±2.38aC	7152.85±336.46aAB	2.59±0.12aB
	W2N0	412.78±44.13bAB	106.02±7.84dBC	−185.04±12.35dAB	4367.27±466.03cAB	2.41±0.12bB
	W2N1	449.83±37.38bB	161.37±19.70cB	−135.20±14.56cB	4902.33±431.51cB	2.30±0.07bB
	W2N2	527.37±32.64aAB	212.12±17.63bBC	−87.28±7.80bB	5829.22±376.63bB	2.25±0.10bAB
	W2N3	596.87±45.25aAB	271.26±21.82aBC	−34.01±6.12aB	6700.05±513.72aAB	2.75±0.04aB
	W3N0	391.21±31.05cB	96.24±11.36dC	−172.01±15.14dA	4128.40±345.60bB	2.78±0.15abA
	W3N1	429.06±41.95bcB	143.33±10.03cB	−102.21±10.68cA	4654.29±449.76bB	2.57±0.06bcA
	W3N2	500.59±30.59abB	193.78±17.92bC	−59.47±4.82bA	5518.86±356.12aB	2.43±0.17cB
	W3N3	562.69±47.75aB	249.92±21.77aC	−11.49±1.11aA	6306.07±537.23aB	2.99±0.09aA
	ANOVA					
	W	**	**	**	**	**

N	**	**	**	**	**
W×N	ns	ns	ns	ns	ns

Note: W, N, and W×N represent the main effects of irrigation gradient, nitrogen application level, and their interaction, respectively, * and ** indicate significance at the $P<0.05$ and $P<0.01$ levels, respectively, and ns indicates no significance. Lowercase letters indicate differences among nitrogen application rates under the same irrigation level, uppercase letters indicate differences among irrigation gradients under the same nitrogen application level.
